# SnO_2_ Nanosheet/Nanoparticle Detector for the Sensing of 1-Nonanal Gas Produced by Lung Cancer

**DOI:** 10.1038/srep10122

**Published:** 2015-05-20

**Authors:** Yoshitake Masuda, Toshio Itoh, Woosuck Shin, Kazumi Kato

**Affiliations:** 1 National Institute of Advanced Industrial Science and Technology (AIST), 2266-98 Anagahora, Shimoshidami, Moriyama-ku, Nagoya 463-8560, Japan

## Abstract

A sensor has been developed for detecting 1-nonanal gas present in the breath of lung cancer patients by combining SnO_2_ nanosheets with SnO_2_ nanoparticles and noble metal catalysts. A significant change in the electrical resistance of this sensor was observed with increasing 1-nonanal gas concentration; the resistance decreased by a factor of 1.12 within the range of 1 to 10 ppm at 300 °C. The recovery of the sensor’s resistance after detecting 1-nonanal gas concentrations of 0.055, 0.18, 1, and 9.5 ppm was determined to be 86.1, 84.2, 80.4 and 69.2%, respectively. This high sensitivity is attributed to the accelerated oxidation of 1-nonanal molecules caused by the (101) crystal faces of the SnO_2_ nanosheets and should provide a simple and effective approach to the early detection of lung cancer.

Early detection is the best defence against lung cancer and is therefore of vital importance in the development of a simple exhaled breath analysis system for lung cancer detection^1^,^2^,^3^,^4^. It is known that the breath of lung cancer patients contains traces of 1-nonanal gas^5^, which as a by-product of the destruction of cell membranes, increases in concentration in relation to the damage caused by smoking^6^. Although this raises the possibility of creating 1-nonanal gas sensors for on-site monitoring in households for early detection, the difficulty of oxidizing a large molecule such as 1-nonanal creates a significant hurdle to the development of highly sensitive detector materials. A potential solution to this lies in the use of SnO_2_ particulate films, which have been successfully used in sensors for volatile organic compounds^7^,^8^,^9^,^10^,^11^ and combined with nanocarbon materials^12^,^13^ such as carbon nanotubes and graphene to create electronic gas sensors capable of functioning at room temperature. Indeed, the authors of this paper have recently reported a 1-nonanal gas sensor based on SnO_2_^14^, in which concentration is determined from the change in the resistance of the sensor that is caused by 1-nonanal oxidizing to CO_2_ and H_2_O in the presence of a noble metal catalyst, thereby reducing the SnO_2_. The resulting movement of electrons from aldehyde groups of 1-nonanol back into the conduction band of SnO_2_^15^ has the effect of reducing the depth of the space-charge region^14^, which along with the valence of the Sn, influences the sensitivity and response of the sensor. Thus, although a limited change in resistance has so far been achieved, greater optimisation of the structure should greatly increase the sensitivity of this detector material. To this end, an improved SnO_2_ nanosheet detector for the detection of lung cancer^16^ has been developed that combines SnO_2_ nanosheets with SnO_2_ nanoparticles and noble metal catalysts, the sensing properties of which are herein discussed.

## Methods

**Figure 1:**
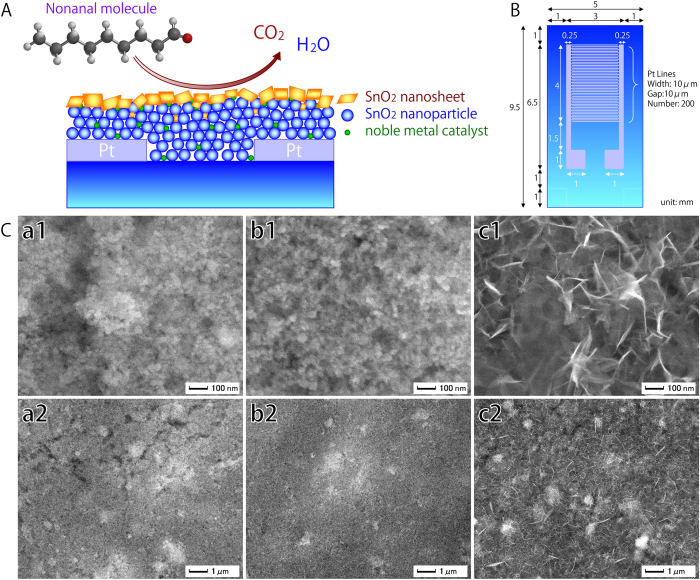
A: Schematic view of the reaction of a 1-nonanal molecule on a sensor consisting of SnO_2_ nanosheets, SnO_2_ nanoparticles, and noble metal catalysts. B: Schematic showing the layout of Pt inter-digital electrodes used in the sensor. C: FE-SEM images of the surface morphology of SnO_2_ particulate films with SnO_2_ nanosheets. (**a1,a2**) SnO_2_ particulate film. (**b1,b2**) SnO_2_ particulate film with SnO_2_ nanosheets formed after 20 min immersion in SnF_2_ solution. (**c1,c2**) SnO_2_ particulate film with SnO_2_ nanosheets formed after 6 h immersion.

To create the 1-nonanal gas sensor, a silicon oxide layer 1000 nm in thickness was first thermally formed on a silicon substrate. Onto this, LaAlO_3_ layers several tens of nanometres in thickness were formed, followed by 300 nm-thick Pt inter-digital electrodes. To create the particulate SnO_2_ film containing noble metal catalysts, Pt, Au and Pd nanoparticles were first mixed with SnO_2_ particles several tens of nanometres in diameter to a SnO_2_:Pt:Au:Pd ratio of 97:1:1:1 by weight. These mixed particles were heated at 400 °C for 2 h, and then mixed with ethyl cellulose to form a colloidal solution that was pasted onto the substrates. After drying at 80 °C for 2 h, these were heated at 500 °C for 2 h to create a 3000 nm-thick SnO_2_ particulate film. This was exposed to ultraviolet light for 20 min under vacuum using a low-pressure mercury lamp (PL16-110, SEN Lights Co.) to create a hydrophilic surface. Finally, it was immersed in an aqueous solution^17^ containing SnF_2_ (5 mM) at 90 °C for 6 h to create a top layer of SnO_2_ nanosheets, then washed with running water and dried by blowing air. Fig. 1A provides a schematic view of the resulting structure. For comparison, nanosheet layers were also produced by 20 min, which has been demonstrated in a previous study^18^ to produce nanosheets of about 10–20 nm in width, 100 nm in length, and 5 nm in thickness; increasing to 5–10 nm in thickness and about 100–1600 nm in plane size after 24 h^19^.

A fine parallel structure of comb-shaped Pt electrodes was used to increase sensitivity (Fig. 1B).

The surface morphology of the sensors was observed by field emission scanning electron microscopy (FE-SEM; JSM-6335FM, JEOL Ltd.), while their cross-sectional structure was observed using transmission electron microscopy (TEM; JEM-2100F, HRPP, 200 kV, JEOL Ltd.). The individual SnO_2_ nanosheets and SnO_2_ particles were observed with scanning-TEM-high-angle-annular-dark-field (HAADF), energy dispersive X-ray spectrometry (EDS, JED-2300T, DRY SD100GV, JEOL Ltd.), and electron energy-loss spectroscopy (EELS, Quantum ER, Gatan, Inc.). The structure of 1-nonanal was calculated at the Hartree–Fock/3-21G(*) level using molecular modelling and computational chemistry (Spartan, Wavefunction, Inc.).

The sensor’s ability to detect 1-nonanal gas was evaluated by first measuring its inter electrode resistance in flowing air over a period of 180 s to give a value hereafter defined as Ra (ohm). The flowing gas was then changed to 1-nonanal, and after 300 s, the value Rg (ohm) was recorded. Air was then re-introduced as the flowing gas, and at 600 s, the R_600_ (ohm) value was recorded. The recovery ratio of the sensor was then calculated as (R_600_-Rg)/(Ra-Rg). This process was repeated for 1-nonanal gas concentrations of 0.055, 0.18, 1, and 9.5 ppm.

## Results and Discussion

**Figure 2:**
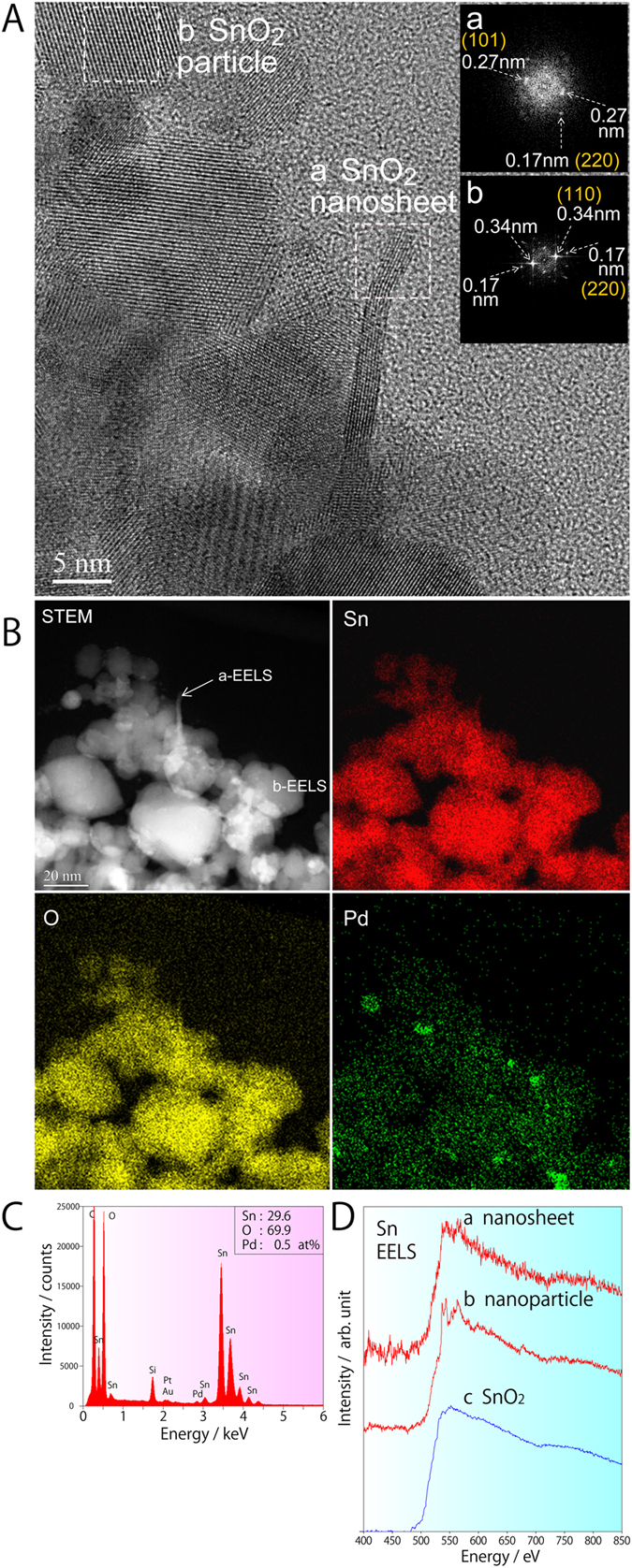
A: Cross-sectional TEM image of a SnO_2_ particulate film with SnO_2_ nanosheets created by 20 min immersion in SnF_2_ solution (NS(20 min)/PF). (**a**) SnO_2_ nanosheet and its FFT power spectrum. (**b**) SnO_2_ particle and its FFT power spectrum. B: Cross-sectional STEM-HAADF image of NS(20 min)/PF together with EDS element maps of Sn, O, and Pd. C: EDS spectrum of the film showing its chemical ratio. D: EELS spectra of NS(20 min)/PF. (**a**) SnO_2_ nanosheet in the a-EELS area marked in Fig. 2B. (b) SnO_2_ nanoparticle in the b-EELS in Fig. 2B. (c) SnO_2_ standard from the EELS-Atlas database.

The bare SnO_2_ particulate film was found through FE-SEM analysis to have a porous surface structure (Fig. 1C,a1), but showed little change after immersion for 20 min due to the small size of the SnO_2_ nanosheets (Fig. 1C,b1). After 6 h immersion, however, large SnO_2_ nanosheets measuring 100 nm in-plane and about 1–5 nm in thickness could be clearly observed on the particulate films (Fig. 1C,c1). These created a network between the SnO_2_ particles, the noble metal particles and the nanosheets themselves. Subsequent TEM observation revealed that the SnO_2_ nanosheets were directly formed on the SnO_2_ particulate film (Fig. 2A), as they were aligned parallel to the sheet surface. The continuous lattice fringe of the nanosheet also suggests that single crystals were formed. The fast Fourier transform (FFT) spectrum of the nanosheet marked as area “a” in the TEM image revealed lattice spacings of 0.27 and 0.17 nm (Fig. 2A,a), which were assigned to the (101) and (220) planes of SnO_2_, respectively. The fact that the strong 0.17 nm FFT spots were aligned perpendicular to the in-plane direction of the nanosheet suggests that the large flat planes of the nanosheets were formed on the (101) crystal faces of SnO_2_. The SnO_2_ particle in area “b” exhibited clear lattice fringes and lattice spacings of 0.34 and 0.17 nm (Fig. 2A,b), the former being assigned to the (110) plane of SnO_2_.

To obtain clearer information on the chemical composition rather than crystal structure, STEM-HAADF was used to observe the shapes and positions of the various sensor components along with the distribution of Sn, O and Pd (Fig. 2B). The Sn and O concentrations differentiate the SnO_2_ nanosheets/particles from the Pd catalyst, allowing the ratio of Sn:O:Pd to be estimated at 29.6:69.9:0.5. The Pt and Au, however, had only very tiny peaks in the EDS spectrum (Fig. 2C), indicating that greater control over the addition and distribution of these additives is needed to maximise the sensing properties of the detector. The EELS spectra obtained from the SnO_2_ nanosheets and SnO_2_ nanoparticles (Fig. 2D, a or b) were both similar to the spectrum of SnO_2_ in the EELS-Atlas database (Fig. 2D,c), indicating that they contained quadrivalent tin.

**Figure 3:**
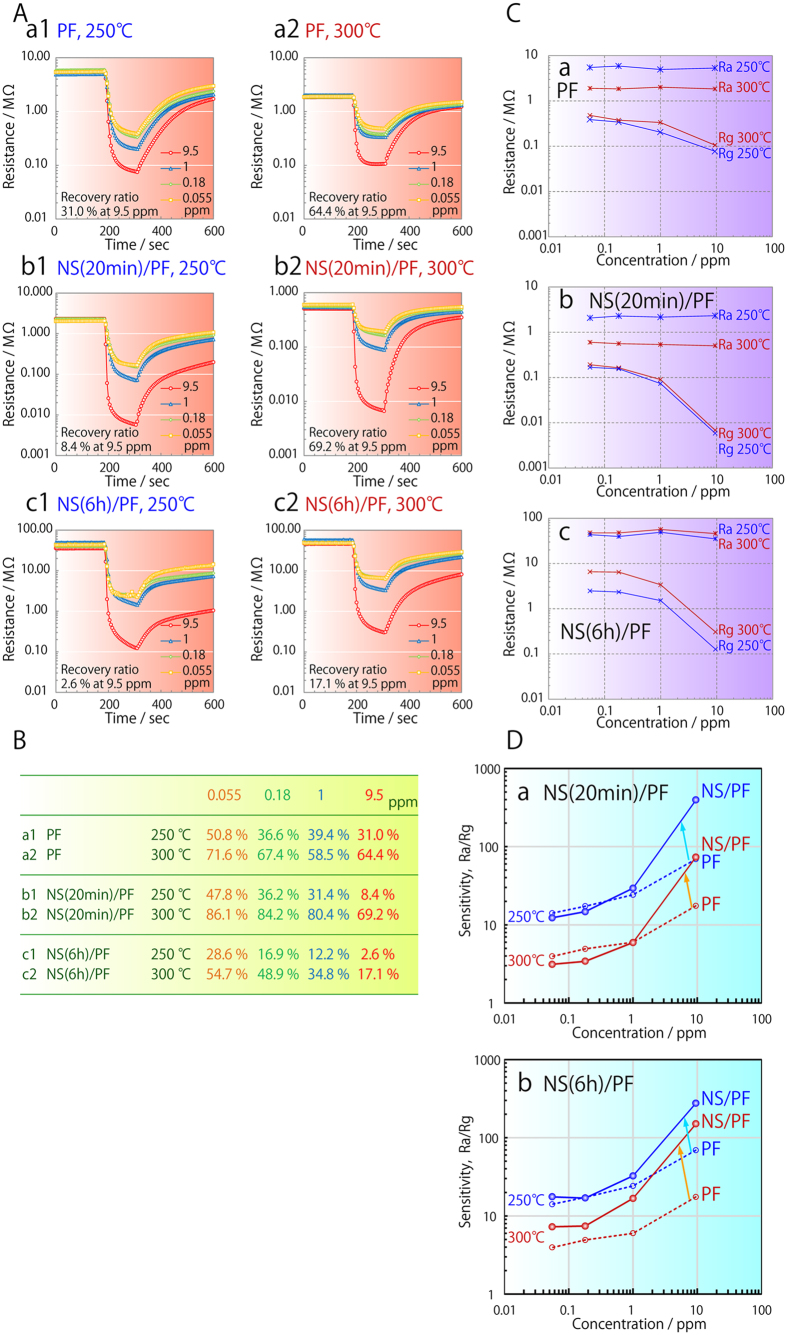
A: Response curves of (**a1,a2**) a particulate film (PF), (**b1,b2**) a particulate film with nanosheets produced by 20 min immersion in SnF_2_ solution (NS(20 min)/PF) and (**c1,c2**) a particulate film with nanosheets produced by 6 h immersion (NS(6 h)/PF). Concentration of 1-nonanal gas was 0.055, 0.18, 1 or 9.5 ppm, and this was evaluated at (**a1,b1,c1**) 250 °C or (**a2,b2,c2**) 300 °C. B: Recovery ratio of the different particulate films. C: Change in resistance as a function of 1-nonanal gas concentration. Ra and Rg of (**a**) PF, (**b**) NS(20 min)/PF and (**c**) NS(6 h)/PF at (blue lines) 250 °C or (red lines) 300 °C. D: Change in sensitivity with 1-nonanal gas concentration for (**a**) NS(20 min)/PF (solid circles and lines) and PF (open circles and dotted lines), and (**b**) NS(6 h)/PF (solid circles and lines) and PF (open circles and dotted lines) at (blue lines) 250 °C and (red lines) 300 °C.

The recovery ratio of sensors consisting solely of SnO_2_ particulate film (PF) was about 31.0% at a 1-nonanal gas concentration of 9.5 ppm and a temperature of 250 °C. After immersion for 20 min or 6 h in SnF_2_ solution (NS(20 min)/PF and NS(6 h)/PF), this dropped to 8.4 and 2.6%, respectively. However, when the temperature was increased to 300 °C, the recovery ratios of PF, NS(20 min)/PF and NS(6 h)/PF were significantly improved to 64.4, 69.2 and 17.1%, respectively, due to the accelerated removal of adsorbed 1-nonanal molecules; residual adsorbed 1-nonanal molecules on NS(6 h)/PF being responsible for its much lower recovery ratio. Significantly, the recovery ratio of NS(20 min)/PF was consistently higher than that of PF in any lean 1-nonanal gas at 300 °C (Fig. 3B, Table); reaching ratios of 86.1, 84.2 and 80.4% in 1-nonanal gas concentrations of 0.055, 0.18 and 1 ppm, respectively. As expected, the Rg of the sensors also decreased with increasing 1-nonanal gas concentration (Fig. 3C) due to the reduction of SnO_2_; however, in the concentration range of 1 to 10 ppm, a far more drastic decrease was seen in the films with nanosheets (Fig. 3C,b,c) than in PF (Fig. 3C,a). This indicates that the SnO_2_ nanosheets have a much greater ability to oxidize 1-nonanal molecules, which can be attributed to their high surface area and characteristic crystal faces.

A decrease in Ra with temperature was observed in the case of PF (Fig. 3C,a) and NS(20 min)/PF (Fig. 3C,b), which can explained by an increase in carrier concentration due to the fact that SnO_2_ is a known semiconductor. This carrier concentration was not affected by temperature in 1-nonanal gas, however, as the reduction of SnO_2_ has a much greater influence. The Ra of NS(6 h)/PF, on the other hand, was not affected by temperature (Fig. 3C,c), although its Rg slightly increased. Given that NS (6 h)/PF contained a greater number of nanosheets than NS(20 min)/PF, it is possible that this may be influenced by the crystallinity, crystal faces, valence, and composition of the nanosheets, but further investigation of the charge transport mechanism is needed to fully understand this connection.

As both the Ra and Rg of NS(20 min)/PF at 250 and 300 °C (Fig. 3C,b) were lower than those of PF at low 1-nonanol concentrations (Fig. 3C,a), it would appear that the nanosheets do provide an effective electrical conduit. The behaviour of the NS(6 h)/PF is very different though, in that its Ra and Rg at 250 °C are slightly reduced at low 1-nonanol concentrations (Fig. 3C,c), but are increased at 300 °C. This would seem to suggest that fine cracks form in the nanosheet layer of NS/PF(20 min) at higher temperatures, thereby increasing its resistance. In either event, the drastic increase in Ra/Rg at 1-nonanal gas concentrations of 1 to 10 ppm clearly demonstrates that the sensitivity of the sensors was successfully enhanced by SnO_2_ nanosheets (Fig. 3D,a,b). This improved the slope of the Ra/Rg curve from 0.47 and 0.48 at 250 and 300 °C in the case of PF, to 0.95 and 0.98 with NS(6 h)/PF. Further improvement to 1.15 and 1.12 was achieved in the case of NS(20 min)/PF. The fact that this improvement in sensitivity becomes more pronounced at higher 1-nonanal gas concentrations (Fig. 3D) suggests that PF alone is insufficient to oxidise such a high concentration of gas.

Nanosheets formed on substrates free of tin were subsequently used to obtain a clearer picture of their specific effects and allow precise X-ray diffraction (XRD) analysis of their structure. The results showed that although the as-prepared nanosheets were predominantly composed of SnO_2_, they also exhibited very weak diffraction peaks for SnO and other related materials. Following heat treatment, both the crystallinity and crystallite size of SnO_2_ increased, while the weaker peaks disappeared. Since the SnO and related materials would presumably have an influence on the charge transport and electrical conductivity, and therefore the sensing properties, this disappearance warrants further investigation to clarify the mechanism behind it. The sensing properties of this unique nanosheet-based sensor can be fully optimised only if there is precise control over these other phases.

The SnO_2_ nanosheets were found through TEM analysis to have large, flat (101) faces, which is the second most stable surface of SnO_2_ after the (110) plane. In contrast, the SnO_2_ particulate films tend to have a mixture of several crystal faces, most of which have a much lower gas-sensing ability^20^. Density-function theory and scanning tunnelling microscopy were therefore performed to clarify the ease with which the (101) surface reversibly switches with a change in atmosphere. The precise mechanism has been previously described^20^, but essentially is based on the premise that the (101) surface is not flat on an atomic scale, and so the removal of O ions changes it from an SnO_2_ surface to a conductive SnO surface. In contrast, the SnO_2_ (110) surface has no analogous arrangement of ions that satisfies the Sn^2+^ oxidation state, and so requires complex reconstruction. In short, this means that the (101) surface can more readily switch from stoichiometric insulating Sn^4+^O_2_^2−^ to reduced conductive Sn^2+^O^2−^, thus making it the better suited to improving the sensitivity of gas detection.

## Conclusions

A 1-nonanal gas sensor has been successfully developed based on a SnO_2_ nanostructured detector, in which a combination of SnO_2_ nanosheets with (101) crystal faces, SnO_2_ nanoparticles and noble catalysts is used to accelerate the oxidation of 1-nonanal molecules. The sensor has been demonstrated to have a dramatically increased sensitivity at concentrations of 1 to 10 ppm, with the slope of its Ra/Rg curve reaching 1.12 at 300 °C. Its recovery ratio of 86.1, 84.2, 80.4 and 69.2% at 1-nonanal gas concentrations of 0.055, 0.18, 1 and 9.5 ppm, respectively, also represents a significant improvement over sensors based on SnO_2_ nanoparticles alone. This indicates that more precise control over the nanostructure of SnO_2_-based sensors can significantly improve their sensitivity, and therefore presents a viable approach to developing simple and highly sensitive 1-nonanal gas sensors for the early detection of lung cancer.

## Additional Information

**How to cite this article**: Masuda, Y. *et al*. SnO_2_ Nanosheet/Nanoparticle Detector for the Sensing of 1-Nonanal Gas Produced by Lung Cancer. *Sci. Rep.* **5**, 10122; doi: 10.1038/srep10122 (2015).
